# Triple Versus Double Therapy for the Treatment of Severe Infections Caused by Carbapenem-Resistant Enterobacteriaceae: A Systematic Review and Meta-Analysis

**DOI:** 10.3389/fphar.2019.01673

**Published:** 2020-01-30

**Authors:** Lei Wang, Xiang Tong, Jizhen Huang, Li Zhang, Dongguang Wang, Man Wu, Tao Liu, Hong Fan

**Affiliations:** Department of Respiratory and Critical Care Medicine, West China Hospital/West China School of Medicine, Sichuan University, Chengdu, China

**Keywords:** carbapenem-resistant Enterobacteriaceae, infection, therapy, survival, meta-analysis

## Abstract

**Introduction:**

The role of combination treatment in the management of carbapenem-resistant Enterobacteriaceae infections (CRE) is still unclear. There have been no meta-analysis comparing the efficiency of triple therapy in treating CRE infections with that of double therapy. In this perspective, we conducted a meta-analysis to clarify whether triple therapy is superior to double therapy in treating patients with CRE infections.

**Methods:**

We performed a systematic review, using PubMed and Embase without any restrictions until October 2019. Risk ratio (RR) with 95% CI were pooled to evaluate the effect of intervention.

**Results:**

A total of 33 studies with 1,441 subjects were identified. Pooled analysis showed that triple therapy was not associated with a reduced mortality compared with double therapy (HR 0.99 95% CI 0.85–1.14, P = 0.85).

**Conclusions:**

This meta-analysis suggests that triple therapy is not superior to double therapy in the treatment of patients with CRE infections, although the quality of evidence is generally low based on current literatures. Future well-defined, randomized controlled trials will be required to elucidate the role of triple therapy in the treatment of CRE infections.

## Introduction

The use of carbapenems has led to the surge of carbapenem-resistant Enterobacteriaceae (CRE), which represents a threat to global public health (McLaughlin et al., 2013; van Duin et al., 2013). These isolates that produce carbapenemase are usually resistant to many non-β-lactams classes of antibiotics such as fluoroquinolones, aminoglycosides, and co-trimoxazole (Bratu et al., 2005; Cuzon et al., 2008; Marchaim et al., 2008). Recent data suggests that the prevalence of CRE is increasing across the world (Lee et al., 2016; Logan and Weinstein, 2017). This is particularly worrisome because the infections caused by CRE are associated with a high mortality.

To date, the optimal treatment of CRE infections remains unknown. The treatment of CRE infections is challenging because only few therapeutic options are available, including colistin, tigecycline, aminoglycoside, and carbapenem in selected cases. This situation has forced clinicians to search for optimal combination strategy to maximize bacterial killing. *In vitro* data also showed that combination therapy is associated with various degrees of synergy and increased bactericidal activity compared with monotherapy (Brennan-Krohn et al., 2017). However, many clinical studies have been carried out to evaluate the efficacy of combination therapy in the treatment of CRE infections, with conflicting results. Tumbarello et al. found that patients with bloodstream infections due to Klebsiella pneumoniae carbapenemase-producing K. pneumoniae receiving two or more drugs had a lower 30-day mortality compared with those taking monotherapy (Tumbarello et al., 2012). On the contrary, another retrospective study including 256 patients claimed that combination therapy was not superior to monotherapy for treatment of infections caused by carbapenem-resistant Enterobacteriaceae (Alexander et al., 2017). Moreover, a recent randomized controlled trial including 406 patients observed that colistin plus meropenem was not superior to colistin monotherapy for treatment of infections caused by carbapenem-resistant Gram-negative bacteria (Paul et al., 2018). It is still unclear whether combination therapy could improve the clinical outcome of patients with CRE infections. Previous trials and reviews exclusively focused on the differences between monotherapy and combination therapy in these patients. In fact, many patients in the combination group actually received two or more antibiotic and the clinical difference between triple therapy and double therapy has not yet been systematically reviewed. The primary goal of this systematic review and meta-analysis was to compare triple therapy with double therapy for the treatment of severe infections caused by CRE.

## Methods

### Data Source and Search Strategy

We performed a systematic literature search in the PubMed and Embase without any restrictions up to October 2019. We used the following search strategy (“extensively drug-resistant” or “multidrug drug-resistant” or “carbapenem-resistant” or “carbapenemase” or “carbapenemase producing” or “Klebsiella pneumoniae carbapenemase” or “NDM” or “VIM” or “IMP” or “OXA”) and (“Gram-negative” or “Enterobacteriaceae” or “Escherichia” or “Klebsiella” or “Enterobacter” or “Proteus” or “Serratia” or “Citrobacter” or “Salmonella” or “Shigella”) and (“survival” or “mortality” or “fatality” or “death” or “lethality” or “predictor” or “prognosis”). To identify more pertinent publications, the reference lists of selected articles were also hand searched.

### Inclusion and Exclusion Criteria

Screening of potentially eligible studies was conducted independently by two authors, and disagreement being resolved by consensus. Articles must have evaluated a therapeutic intervention for the treatment of patients with CRE infections. Studies provided clinical outcome regarding the efficiency of triple and double therapy were considered eligible. Triple and double therapy was defined as any three and two antibiotic combination, respectively. The primary outcome was 30-day mortality and if not reported at day 30 we extracted and documented the closest timepoint. Studies were excluded if any of the following existed: (1) essential data could not be extracted from the published articles; (2) studies reporting on the clinical outcomes of patients colonized with carbapenemase-producing Enterobacteriaceae or CRE were excluded; (3) studies were excluded if they were *in vitro* study, study protocol, letter, note, review, commentary, conference abstract, animal, and children study; (4) case reports and case-series including fewer than 10 infected patients were also excluded. For studies that reported the same cohort, only the study with more patients was considered. All analyses were based on previously published studies; thus, no ethical approval and patient consent are required.

### Data Extraction and Risk of Bias Assessment

Two independent reviewers extracted the information from each study and used a predesigned data extraction excel form. If there was a disagreement, a third reviewer further assessed these articles. The following data were extracted from each included study: first author, publication year, study design, type of infection, causative pathogens, susceptibility breakpoints used for carbapenem and data on mortality for patients with CRE infections. Newcastle-Ottawa Scale was used to evaluate the quality of nonrandomized studies (Wells et al.,).

### Data Analysis

The pooled risk ratio (RR) and 95% confidence interval (CI) was calculated for outcome analysis. Heterogeneity across studies was tested by using the I^2^ statistic, which is a quantitative measure of inconsistency across studies (Higgins and Thompson, 2002). If the P < 0.10 and I^2^ > 50%, there was obvious between-study heterogeneity and a random-effects model was used; otherwise, the fixed-effects model was used. Begg’s test as well as the funnel plot were used to evaluate publication bias. Subgroup analysis were performed according to region, study design, causative pathogens, type of infections, susceptibility breakpoints used, and NOS. All statistical analyses were performed with the use of STATA 12.0 (Stata Corp, College Station, Texas, USA). All P less than 0.05 were considered as significant unless otherwise specified.

## Results

### Description of the Search and Selection of Trials

**Figure 1:**
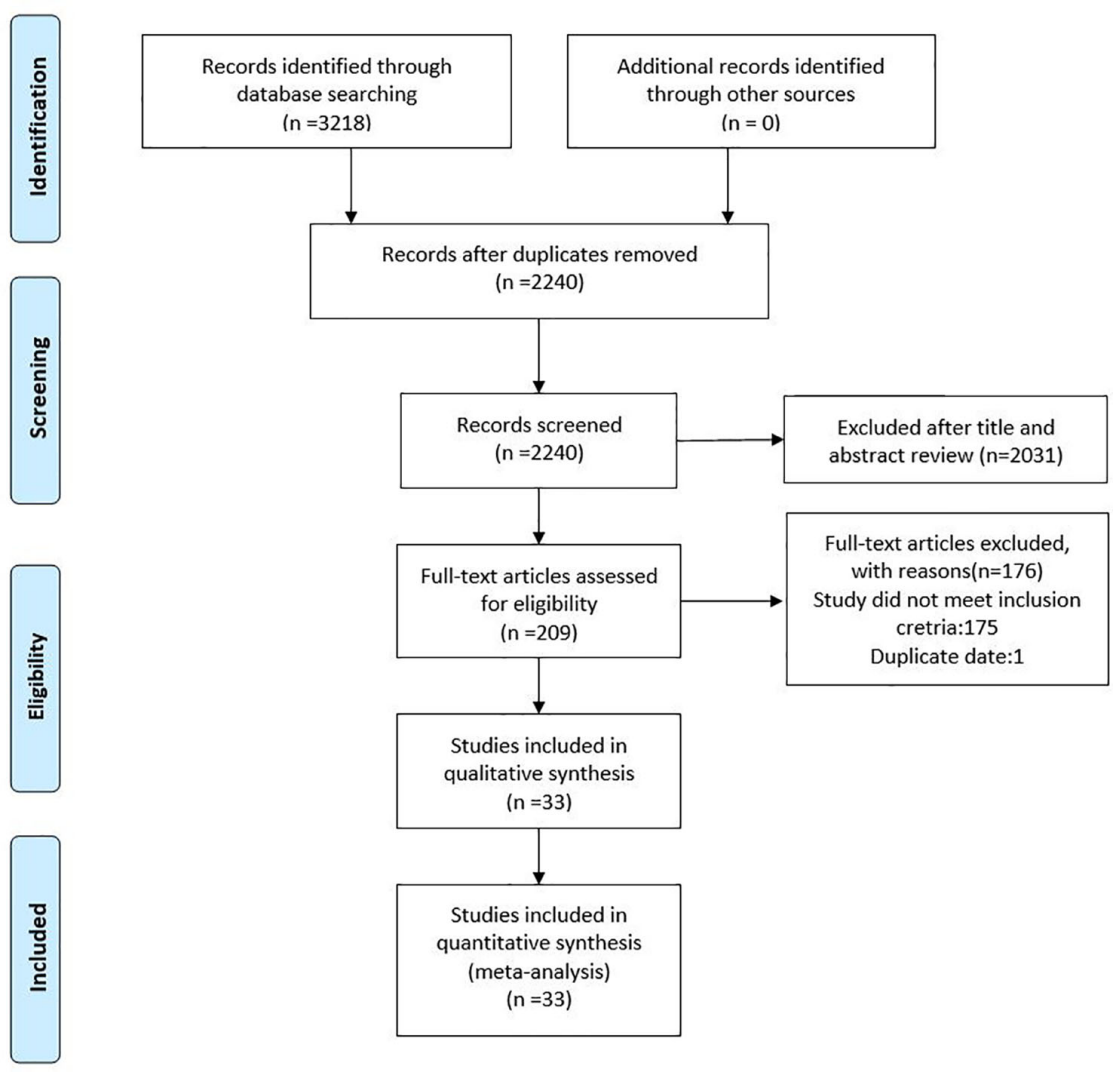
The flow diagram of included and exclude studies.

As shown in **Figure 1**, the literature search strategy initially identified 3,218 articles. 978 studies were excluded because they were duplicated studies. A total of 2,031 articles were removed after initial screening of titles and abstracts. Among the 209 remaining articles, 132 studies provided unclear antibiotic treatment, 17 included patients less than 10 people, 14 did not report clinical outcomes of interest, 12 treated all patients with monotherapy and/or double therapy, and 1 used duplicated data. Hence, a total of 33 eligible studies were included in this current meta-analysis (Souli et al., 2008; Maltezou et al., 2009; Zarkotou et al., 2011; Qureshi et al., 2012; Sanchez-Romero et al., 2012; Navarro-San Francisco et al., 2013; Balandin Moreno et al., 2014; Balkan et al., 2014; Daikos et al., 2014; Kontopidou et al., 2014; Papadimitriou-Olivgeris et al., 2014; Pontikis et al., 2014; Chang et al., 2015; de Oliveira et al., 2015; Freire et al., 2015; Ji et al., 2015; Katsiari et al., 2015; Oliveros Navarro et al., 2015; Tumbarello et al., 2015; Falcone et al., 2016; Satlin et al., 2016; Shields et al., 2016; Trecarichi et al., 2016; de Maio Carrillho et al., 2017; Forcina et al., 2017; Kaur et al., 2017; Liao et al., 2017; Machuca et al., 2017; Wang et al., 2018; Yang et al., 2018; Freire et al., 2019; Medeiros et al., 2019; Xu et al., 2019).

### Characteristics and Risk of Bias Assessment of Included Trials

The basic characteristics of all the included studies are presented in **Table 1**. In total, the 33 retrospective and prospective studies were all published between 2011 and 2019. All studies included patients suffering from severe infections caused by CRE or carbapenemase-producing Enterobacteriaceae (CPE). In particular, two studies focused on oxacillinase (OXA)-48-CPE and Verona integron-encoded metallo-β-lactamase (VIM-1) producing K pneumoniae, respectively. All studies interpreted carbapenem resistance according to the Clinical and Laboratory Standards Institute (CLSI) or European Committee on Antimicrobial Susceptibility Testing (ECAST) except four studies. All articles reported the primary outcomes of interest, 13 reporting 30-day mortality, 7 reporting overall mortality, 7 reporting 28-day mortality, 1 reporting 21-day mortality, 4 reporting 14-day mortality and the remaining 1 reported 7-day mortality. Risk of bias assessment of included trials is summarized in **Table 1**. Overall methodological quality of included studies was moderate, and the NOS scores ranged from five to seven (**Supplementary Table S1**).

**Table 1:** Characteristics of included studies.

Author	Year	Country	Study design	Type of infection	Causative bacteria	Susceptibility breakpoints used	mortality		Outcomes	NOS
							triple therapy	double therapy	
Balkan et al. (2014)	2014	Turkey	Retrospective	Bloodstream infection	OXA-48-like carbapenemase-producing Enterobacteriaceae	EUCAST	3/12	7/12	28-day mortality	6
Chang et al. (2015)	2015	China	Retrospective	Mix	Carbapenem nonsusceptible Enterobacteriaceae	CLSI	1/2	4/8	30-day mortality	6
Daikos et al. (2014)	2014	Greece	Retrospective	Bloodstream infection	Carbapenemase-producing Klebsiella pneumoniae	EUCAST	3/19	25/78	28-day mortality	6
Falcone et al. (2016)	2016	Italy	Retrospective	Mix	Carbapenemase–producing Klebsiella pneumoniae	EUCAST	16/44	13/39	30-day mortality	7
Forcina et al. (2017)	2017	Italy	Retrospective	Bloodstream infection	Carbapenemase-producing Klebsiella pneumoniae	NA	2/8	5/6	30-day mortality	6
Navarro-San Francisco et al. (2013)	2012	Spain	Prospective	Bloodstream infection	OXA-48-carbapenemase-producing Enterobacteriaceae	CLSI	2/2	16/25	Mortality	7
Freire et al. (2015)	2015	Brazil	Retrospective	Mix	KPC-producing Klebsiella pneumoniae	CLSI	8/11	10/22	30-day mortality	6
Freire et al. (2019)	2019	Brazil	Retrospective	Mix	Polymyxin and carbapenem-resistant Enterobacteriaceae	CLSI	4/10	3/8	30-day mortality	6
Souli et al. (2008)	2008	Greece	Retrospective	Mix	KPC-producing Klebsiella pneumoniae	CLSI	3/4	4/8	7-day mortality	6
Ji et al. (2015)	2015	China	Prospective	Mix	KPC-producing Klebsiella pneumoniae	CLSI	2/5	10/40	28-day mortality	7
Katsiari et al. (2015)	2015	Greece	Prospective	Mix	Carbapenemase-producing Klebsiella pneumoniae	CLSI	4/11	5/11	14-day mortality	6
Kaur et al. (2017)	2017	India	Retrospective	Bloodstream infection	Carbapenem and colistin resistant Klebsiella pneumoniae	NA	18/24	13/16	Mortality	7
Kontopidou et al. (2014)	2013	Greece	Retrospective	Mix	Carbapenem-resistant Klebsiella pneumoniae	CLSI	1/5	17/37	Mortality	6
Liao et al. (2017)	2017	China	Retrospective	Mix	Carbapenem-resistant Klebsiella pneumoniae	NA	2/15	13/57	Mortality	7
Maltezou et al. (2009)	2009	Greece	Retrospective	Mix	KPC-producing Klebsiella pneumoniae	CLSI	0/2	2/9	14-day mortality	6
Machuca et al. (2017)	2017	Spain	Prospective	Bloodstream infection	KPC-producing Klebsiella pneumoniae	EUCAST	6/32	12/40	30-day mortality	7
de Maio Carrillho et al. (2017)	2017	Brazil	Prospective	Mix	Carbapenem and colistin resistant Enterobacteriaceae	CLSI	4/10	2/4	Mortality	7
Medeiros et al. (2019)	2018	Brazil	Retrospective	Bloodstream infection	KPC-producing Klebsiella pneumoniae	CLSI	12/31	19/35	30-day mortality	7
Balandin Moreno et al. (2014)	2014	Spain	Retrospective	Mix	VIM-1-producing Klebsiella pneumoniae	CLSI	0/3	4/11	30-day mortality	7
de Oliveira et al. (2015)	2014	Brazil	Retrospective	Bloodstream infection	KPC-producing Enterobacteriaceae	CLSI	13/19	16/36	30-day mortality	7
Papadimitriou-Olivgeris et al. (2014)	2014	Greece	Retrospective	Bloodstream infection	KPC-producing Klebsiella pneumoniae	CLSI	2/10	2/5	30-day mortality	6
Pontikis et al. (2014)	2013	Greece	Prospective	Mix	carbapenemase-producing Klebsiella pneumoniae	CLSI	3/8	2/6	28-day mortality	6
Qureshi et al. (2012)	2012	United States	Retrospective	Bloodstream infection	KPC-producing Klebsiella pneumoniae	CLSI	1/3	1/12	28-day mortality	6
Satlin et al. (2016)	2016	United States	Retrospective	Bloodstream infection	carbapenem-resistant Enterobacteriaceae	CLSI	4/8	6/16	30-day mortality	7
Shields et al. (2016)	2016	United States	Retrospective	Bloodstream infection	Carbapenem-resistant Klebsiella pneumoniae	CLSI	2/5	5/16	30-day mortality	7
Sánchez-Romero et al. (2012)	2011	Spain	Retrospective	Mix	VIM-1-producing Klebsiella pneumoniae	CLSI	0/1	7/11	14-day mortality	6
Trecarichi et al. (2016)	2016	Italy	Prospective	Bloodstream infection	Carbapenem-resistant Klebsiella pneumoniae	NA	27/67	3/29	21-day mortality	6
Tumbarello et al. (2015)	2015	Italy	Retrospective	Mix	KPC-producing Klebsiella pneumoniae	EUCAST	67/217	38/134	14-day mortality	6
Wang et al. (2018)	2019	China	Retrospective	Bloodstream infection	Carbapenem-resistant Enterobacteriaceae	CLSI	1/1	1/19	Mortality	7
Yang et al. (2018)	2018	China	Retrospective	Bloodstream infection	Carbapenem-resistant Klebsiella pneumoniae	CLSI	3/8	4/15	28-day mortality	6
Zarkotou et al. (2011)	2011	Greece	Prospective	Bloodstream infection	KPC-producing Klebsiella pneumoniae	CLSI	0/6	0/14	Mortality	6
Oliveros Navarro et al. (2015)	2014	Colombia	Retrospective	Bloodstream infection	carbapenems-resistant Enterobacteriaceae	CLSI	10/21	21/26	28-day mortality	7
Xu et al. (2019)	2019	China	Retrospective	Mix	KPC-producing Klebsiella pneumoniae	CLSI	5/5	7/7	30-day mortality	5

KPC, Klebsiella pneumoniae carbapenemase; VIM, Verona integron-encoded metallo-β-lactamase; OXA, oxacillinase; CLSI, Clinical and Laboratory Standards Institute; EUCAST, European Committee on Antimicrobial Susceptibility Testing.

### Mortality

**Figure 2:**
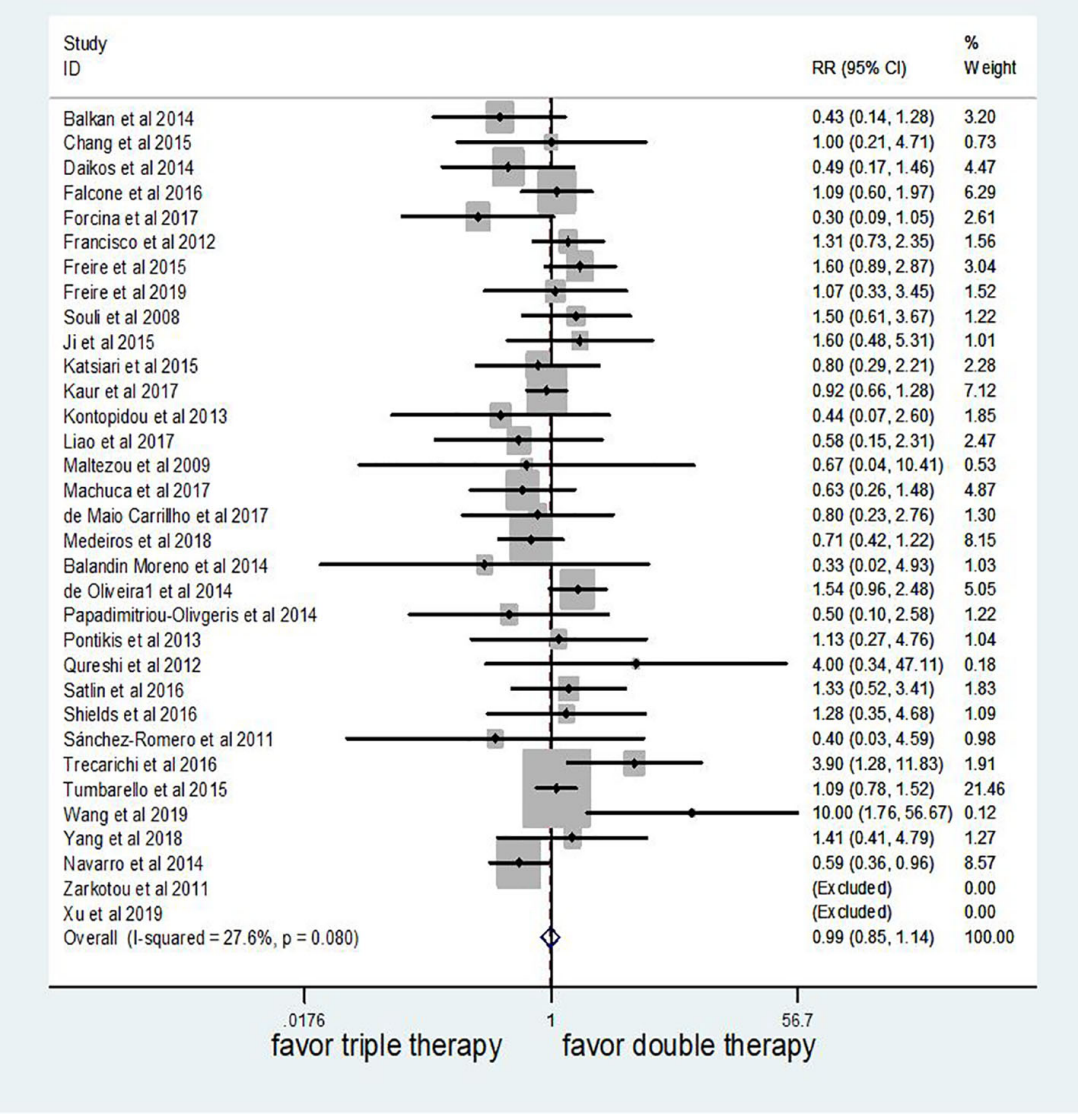
The efficacy of triple therapy, as compared with double therapy, in the treatment of infections due to carbapenem-resistant Enterobacteriaceae (CRE).

Meta-analysis of these studies showed that mortality did not signiﬁcantly differ between triple therapy and double therapy based on fixed effect model (HR 0.99 95% CI 0.85–1.14, P = 0.85; **Figure 2**). The heterogeneity across studies was low (I^2^ = 27.6%, P = 0.08).

The subgroup analysis by study characteristics is shown in **Table 2**. Our meta-analysis showed that the outcome did not differ significantly on the basis of any of the following individual study characteristics: region, study design, type of infection, causative bacteria, susceptibility breakpoints used, outcome, as well as NOS.

**Table 2:** Subgroup analysis according to study characteristics.

Variables	Number of studies	RR (95% CI)	P -value	I^2^ (%)
Region				
North America	3	1.47 (0.72–3.02)	0.29	0
South America	6	0.95 (0.74–1.21)	0.66	5.81
Asia Europe	6	1.05 (0.75–1.48)	0.77	40.9
Europe	16	0.96 (0.78–1.20)	0.74	22.6
Study design				
Retrospective	24	0.94 (0.80–1.09)	0.40	30.4
Prospective	7	1.30 (0.87–1.94)	0.20	26.4
Type of infection				
Bloodstream infection	16	0.95 (0.78–1.15)	0.57	55.7
Mix	15	1.03 (0.83–1.29)	0.78	0
Causative bacteria
Carbapenem-resistant Klebsiella pneumoniae	5	1.50 (0.85–2.63)	0.16	38.9
Carbapenemase-producing Klebsiella pneumoniae	16	0.92 (0.75–1.13)	0.44	4.6
Susceptibility breakpoints used				
CLSI	22	1.00 (0.82–1.22)	0.99	18.8
EUCAST	5	0.91 (0.71–1.18)	0.50	23.5
Outcomes				
30-day mortality	12	0.97 (0.77–1.22)	0.79	22.0
28-day mortality	7	0.71 (0.49–1.02)	0.06	14.9
14-day mortality	6	1.03 (0.75–1.41)	0.87	0
mortality	4	0.91 (0.66–1.26)	0.58	49.6
NOS				
≥7	14	0.93 (0.76–1.12)	0.42	38
<7	17	1.05 (0.84–1.31)	0.68	21.5

RR, risk ratio; CI, confidence interval; CLSI, Clinical and Laboratory Standards Institute; EUCAST, European Committee on Antimicrobial Susceptibility Testing.

### Publication Bias

**Figure 3:**
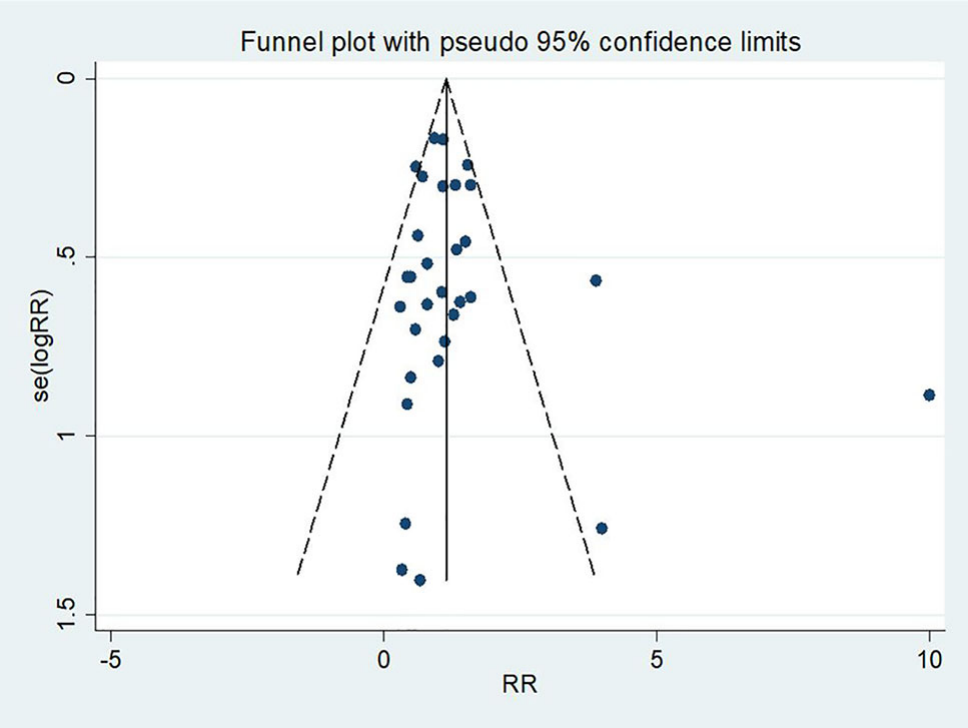
A funnel plot of mortality rate in patients treated with triple therapy compared with that in patients treated with double therapy for infections caused by carbapenem-resistant Enterobacteriaceae (CRE).

No significant statistical bias was detected by Begg’s methods and the funnel plot demonstrated no marked evidence of asymmetry (P = 0.66, **Figure 3**).

## Discussion

To our knowledge, this is the first systematic review and meta-analysis to compare triple versus double antibiotic therapy among patients with CRE infections who have limited treatment options. Our meta-analysis failed to show the superiority of triple over double antibiotic therapy in the treatment of these patients.

There is no doubt that antibiotics have revolutionized medical practice. However, the widespread use of antibiotics has spurred the emergence of CRE. Since the first case was reported in 2001, CRE have spread worldwide (Munoz-Price et al., 2013). Moreover, infections due to CRE are associated with an alarming mortality rate, with an estimated fatality of more than 50% (Falagas et al., 2014). As a result, the US Centers for Disease Control and Prevention (CDC) recognized CRE as one of the three most urgent antimicrobial resistant threats. New therapeutic strategies are desperately needed to address this increasingly important global public health problem. Although we made a comprehensive subgroup analysis, no individual study characteristic was associated with an improved clinical outcome among patients receiving triple antibiotic therapy. This finding is essential because the adoption of triple therapy for CRE might lead to excessive use of antibiotics, resulting in a vicious cycle of antibiotic use and antibiotic resistance. A previous meta-analysis involving 3,627 participants demonstrated that prior antibiotic use such as carbapenem and aminoglycoside was an important risk factor of carbapenem-resistant Klebsiella pneumoniae infection (Liu et al., 2018). Given that roughly 30%–50% of the antibiotic use in hospitals is unnecessary and the limitation of antimicrobial drugs has the potential to reduce the prevalence of CRE, strong antibiotic stewardship policies are urgently needed to curb unnecessary prescribing to ensure more judicious use of antibiotics (Yong et al., 2010; Fleming-Dutra et al., 2016; Tacconelli et al., 2018). Our meta-analysis is in accord with a previous *in vitro* study which observed that adding a third antibiotic did not enhance synergistic effect in multidrug-resistant Klebsiella pneumonia isolates (Stein et al., 2015). It seems plausible that the use of triple therapy might act synergistically to kill bacteria and hopefully improve clinical outcomes. Unfortunately, in our meta-analysis, this supposed synergy failed to translate into improved clinical outcome. One potential explanation for this finding is that triple therapy might have little if any additional pharmacologic effect compared with double therapy. Another potential explanation relates to an unfavorable balance between positive and negative effects of triple therapy. Although there have been cases successfully treated with triple therapy, there is currently no large clinal trial specifically investigating the safety profile of triple therapy among patients with severe infections. Moreover, giving a noncovering antibiotic, especially carbapenem, to patients is not clinically harmless. The added antibiotics might favor the development of Clostridium difficile infection, which is a common healthcare-associated disease worldwide (Vuotto et al., 2018). Therefore, our meta-analysis does not support the routine triple therapy in the management of CRE infections based on currently available evidence.

With the increasing prevalence of CRE, it is urgent for the medical community to develop novel antibiotics. The recent introduction of β-lactamase inhibitors such as avibactam provided alternative treatment options for severe infections due to CRE. In 2015, the US Food and Drug Administration (FDA) approved ceftazidime-avibactam for the treatment of complicated intraabdominal and urinary tract infections (López-Hernández et al., 2017). Another promising agent is plazomicin, a novel aminoglycoside with *in vitro* activity against CRE. However, future large clinical trials are desperately needed to clarify the efficiency of these drugs in the treatment of serious infections due to CRE.

Our study has several strengths. First, this systematic review focuses on a precise clinical question that deserves future research among patients with CRE infections. Second, our study includes a total of 33 retrospective and prospective studies covering 1,441 patients with CRE infections. Third, it takes several important factors into consideration in the analysis of our data, which adds to the robustness of this study. Several limitations should be considered when interpreting our finding. First, although we observed a noninferiority of double therapy in the treatment of CRE infections compared with triple therapy, this conclusion is exclusively from retrospective and prospective studies. Result from nonrandomized controlled trials is susceptible to bias and confounders. Second, the study failed to compare different triple therapies because there are many combinations in terms of triple versus double antibiotic and some articles do not provide detailed information about the antibiotics used, it is unclear whether a specific triple regimen would improve the clinical outcomes of these patients. Finally, the quality of the included studies was not homogeneous, which may originate from differences in study design and data analysis. As a result, the confidence in estimates of effect in this meta-analysis was generally low. Despite these limitations, our meta-analysis has rigorously compared the efficiency of triple therapy in the treatment of patients with CRE infections with that of double therapy.

## Conclusion

In summary, by applying a comprehensive search strategy, we found that triple antibiotic therapy is not superior to double antibiotic therapy in the treatment of patients with CRE infections, although this result requires cautious interpretation. Triple therapy may be a suboptimal choice for infections due to CRE and the optimal treatment of such conditions remains unknown. To date, there have been no randomized controlled trial to address whether triple therapy improves clinical outcomes among patients with infections caused by CRE. Future well-defined, randomized controlled trials will be required to elucidate the role of triple therapy in the treatment of CRE infections.

## Author Contributions

HF conceived the studies. LW, XT, and HJ carried out the literature search and interpretation of the data. LZ and DW helped conduct the analyses. MW and TL assessed the risk of bias. LW wrote the manuscript. All authors approved the final manuscript.

## Funding

This study was supported by the National Key R&D Program of China (2017YFC1309703).

## Conflict of Interest

The authors declare that the research was conducted in the absence of any commercial or financial relationships that could be construed as a potential conflict of interest.
