# Supplementary material for: Triple Versus Double Therapy for the Treatment of Severe Infections Caused by Carbapenem-Resistant Enterobacteriaceae: A Systematic Review and Meta-Analysis
Source: Front Pharmacol. 2020 Jan 30;10:1673. doi: 10.3389/fphar.2019.01673 (PMC7005522; doi:10.3389/fphar.2019.01673)
Supplement: Supplementary file 1 [file Table_1.doc]

Table S1 Quality assessment of included cohort studies

| Author/Year | A | B | C | D | E | F | G | H | J | Total |
| --- | --- | --- | --- | --- | --- | --- | --- | --- | --- | --- |
| Balkan et al 2014 | 1 | 1 | 1 | 1 | 0 | 0 | 1 | 1 | 0 | 6 |
| Chang et al 2015 | 1 | 1 | 1 | 1 | 0 | 0 | 1 | 1 | 0 | 6 |
| Daikos et al 2014 | 1 | 1 | 1 | 1 | 0 | 0 | 1 | 1 | 0 | 6 |
| Falcone et al 2016 | 1 | 1 | 1 | 1 | 0 | 0 | 1 | 1 | 1 | 7 |
| Forcina et al 2017 | 1 | 1 | 0 | 1 | 0 | 0 | 1 | 1 | 1 | 6 |
| Francisco et al 2012 | 1 | 1 | 1 | 1 | 0 | 0 | 1 | 1 | 1 | 7 |
| Freire et al 2015 | 1 | 1 | 1 | 1 | 0 | 0 | 1 | 1 | 0 | 6 |
| Freire et al 2019 | 1 | 1 | 0 | 1 | 0 | 0 | 1 | 1 | 1 | 6 |
| Souli et al 2010 | 1 | 1 | 1 | 1 | 0 | 0 | 1 | 0 | 1 | 6 |
| Ji et al 2015 | 1 | 1 | 1 | 1 | 0 | 0 | 1 | 1 | 1 | 7 |
| Katsiari et al 2015 | 1 | 1 | 1 | 1 | 0 | 0 | 1 | 0 | 1 | 6 |
| Kaur et al 2017 | 1 | 1 | 1 | 1 | 0 | 0 | 1 | 1 | 1 | 7 |
| Kontopidou et al 2013 | 1 | 1 | 1 | 1 | 0 | 0 | 1 | 1 | 0 | 6 |
| Liao et al 2017 | 1 | 1 | 1 | 1 | 0 | 0 | 1 | 1 | 1 | 7 |
| Maltezou et al 2009 | 1 | 1 | 1 | 1 | 0 | 0 | 1 | 0 | 1 | 6 |
| Machuca et al 2017 | 1 | 1 | 1 | 1 | 0 | 0 | 1 | 1 | 1 | 7 |
| de Maio Carrillho et al 2017 | 1 | 1 | 1 | 1 | 0 | 0 | 1 | 1 | 1 | 7 |
| Medeiros et al 2018 | 1 | 1 | 1 | 1 | 0 | 0 | 1 | 1 | 1 | 7 |
| Balandin Moreno et al 2014 | 1 | 1 | 1 | 1 | 0 | 0 | 1 | 1 | 1 | 7 |
| de Oliveira1 et al 2014 | 1 | 1 | 1 | 1 | 0 | 0 | 1 | 1 | 1 | 7 |
| Papadimitriou-Olivgeris et al 2014 | 1 | 1 | 1 | 1 | 0 | 0 | 1 | 1 | 0 | 6 |
| Pontikis et al 2013 | 1 | 1 | 1 | 1 | 0 | 0 | 1 | 1 | 0 | 6 |
| Qureshi et al 2012 | 1 | 1 | 1 | 1 | 0 | 0 | 1 | 1 | 0 | 6 |
| Satlin et al 2016 | 1 | 1 | 1 | 1 | 0 | 0 | 1 | 1 | 1 | 7 |
| Shields et al 2016 | 1 | 1 | 1 | 1 | 0 | 0 | 1 | 1 | 1 | 7 |
| Sánchez-Romero et al 2011 | 1 | 1 | 1 | 1 | 0 | 0 | 1 | 0 | 1 | 6 |
| Trecarichi et al 2016 | 1 | 1 | 1 | 1 | 0 | 0 | 1 | 0 | 1 | 6 |
| Tumbarello et al 2015 | 1 | 1 | 1 | 1 | 0 | 0 | 1 | 0 | 1 | 6 |
| Wang et al 2019 | 1 | 1 | 1 | 1 | 0 | 0 | 1 | 1 | 1 | 7 |
| Yang et al 2018 | 1 | 1 | 1 | 1 | 0 | 0 | 1 | 1 | 0 | 6 |
| Zarkotou et al 2011 | 1 | 1 | 1 | 1 | 0 | 0 | 1 | 1 | 0 | 6 |
| Navarro et al 2014 | 1 | 1 | 1 | 1 | 0 | 0 | 1 | 1 | 1 | 7 |
| Xu et al 2019 | 1 | 1 | 0 | 1 | 0 | 0 | 1 | 1 | 0 | 5 |

A, representativeness of the exposed cohort; B, selection of the non-exposed cohort; C, Ascertainment of exposure; D, demonstration that outcome of interest was not present at start of study; E, study controls for the most important factors; F, study controls for any additional factor; G, assessment of outcome; H, was follow-up long enough for outcomes to occur; J, adequacy of follow up of cohorts;
